# Supplementary material for: Bisphenol A Exposure and Sperm ACHE Hydroxymethylation in Men
Source: Int J Environ Res Public Health. 2019 Jan 8;16(1):152. doi: 10.3390/ijerph16010152 (PMC6339044; doi:10.3390/ijerph16010152)
Supplement: Supplementary file 1 [file ijerph-16-00152-s001.pdf]

Supplemental tables

Table S1. The differences of pre-shift and post-shift BPA levels in BPA-exposed group.

	N	Detection rate%	Median	Minimum	Maximum	Percentiles		*t*	*p*
						5th	95th		
Pre-shift	74	96.3	89.96	LOD	217060.90	0.57	12862.53		
Post-shift	74	100	180.59	0.75	264219.40	1.45	23979.51	3.62	0.001

Table S2. The difference of urine BPA in men with different education category.

(I) edu	(J) edu	Mean Difference (I-J)	Standard Error	*p*
≤Middle school	High school	1.56*	0.66	0.019
	≥College	2.63*	0.83	0.002
High school	≤Middle school	-1.56*	0.66	0.019
	≥College	1.07	0.73	0.146
≥College	≤Middle school	-2.63*	0.83	0.002
	High school	-1.07	0.73	0.146

*α=0.05.
